# Data on the phospholipid fatty acyl composition of retroperitoneal white adipose tissue in ad libitum fed and fasted mice

**DOI:** 10.1016/j.dib.2016.02.078

**Published:** 2016-03-04

**Authors:** Kristin A. Marks, Phillip M. Marvyn, Juan J. Aristizabal Henao, Ryan M. Bradley, Ken D. Stark, Robin E. Duncan

**Affiliations:** University of Waterloo, Department of Kinesiology, Faculty of Applied Health Sciences, 200 University Avenue W., BMH1110, Waterloo, Ontario, Canada N2L 3G1

## Abstract

Data are presented on the fatty acyl composition of phospholipid from retroperitoneal white adipose tissue of female mice that were either given ad libitum access to food or fasted for 16 h overnight prior to sacrifice. Our data show that total adipose phospholipid concentrations were more than 2-fold higher in the fasted animals compared with the fed animals (33.48±7.40 versus 16.57±4.43 μg phospholipid fatty acids/100 mg tissue). Concentrations of several individual phospholipid fatty acyl species, including palmitic acid (16:0), vaccenic acid (18:1n-7), linoleic acid (18:2n-6), dihomo-gamma-linolenic acid (20:3n-6), arachidonic acid (20:4n-6), eicosapentaenoic acid (20:5n-3) and docosahexaenoic acid (22:6n-3), as well as total phospholipid saturated fatty acids, n-6 polyunsaturated fatty acids and n-3 polyunsaturated fatty acids, were significantly higher in adipose tissue from the fasted animals compared with the fed animals. However, when the relative abundance of phospholipid fatty acyl species was analyzed, only 20:4n-6 was specifically enriched (by ~2.5-fold) in adipose phospholipid with fasting.

**Specifications Table**

**Table:** 

Subject area	*Biology, chemistry*
More specific subject area	*Lipid biochemistry*
Type of data	*Gas chromatography, Table*
How data was acquired	*Varian 3900 gas chromatograph equipped with a DB-FFAP* 15 m*,* 0.10 mm *i.d.,* 0.10 lm *film thickness, nitroterephthalic acid modified, polyethylene glycol, capillary column*
Data format	*Analyzed*
Experimental factors	*Mice were allowed ad libitum access to food, or were fasted for* 16 h*, prior to sacrifice*
Experimental features	*Adipose phospholipid were isolated by thin layer chromatography and quantified by gas chromatography*
Data source location	*University of Waterloo, Waterloo, Ontario, Canada*
Data accessibility	*Data is provided within this article*

**Value of the data**

• These data are of value to researchers interested in adipose tissue biology.
• These data are of value to researchers interested in phospholipid metabolism.
• These data are of value to researchers studying bioactive polyunsaturated fatty acids such as arachidonic acid and docosahexaenoic acid.
• Data from fasted compared to non-fasted animals are of value to researchers for use in planning optimal metabolic conditions for experiments**.**

## 1 Data

During fasting, the total concentration of adipose phospholipids increased by ~2-fold (Table 1). Concentrations of several individual phospholipid fatty acyl species, including palmitic acid (16:0), vaccenic acid (18:1n-7), linoleic acid (18:2n-6), dihomo-gamma-linolenic acid (20:3n-6), arachidonic acid (20:4n-6), eicosapentaenoic acid (20:5n-3) and docosahexaenoic acid (22:6n-3), as well as total phospholipid saturated fatty acids, n-6 polyunsaturated fatty acids and n-3 polyunsaturated fatty acids, were significantly higher in adipose tissue from the fasted animals compared with the fed animals (Table 1). However, when the proportion of different fatty acyl species within adipose phospholipid were considered relative to the total phospholipid fatty acyl pool, these data only demonstrate a significant decrease in abundance of docosapentaenoic acid (22:5n-6), and a significant increase in abundance of 20:4n-6 (Table 2).

## 2 Experimental design, materials and methods

### 2.1 Animals

All animal procedures were approved by the University of Waterloo Animal Care Committee and were in accordance with the guidelines of the Canadian Council on Animal Care. Female C57Bl/6J mice, aged 12–16 weeks, were housed in the Central Animal Housing Facility at the University of Waterloo with ad libitum access to a low-fat diet (D12450H, Research Diets Inc., New Jersey, U.S.A.) and water, at a temperature of 21±1 °C on a 12:12-h light-dark cycle. The fatty acid profile of the diet has been reported previously [1]. Prior to sacrifice by cervical dislocation, animals were either fasted overnight for 16 h or maintained with ad libitum access to food. Adipose tissue was collected and immediately frozen in liquid nitrogen, and stored at −80 °C until lipid analysis [1].

### 2.2 Fatty acid composition

Adipose lipids were extracted with 2:1 chloroform:methanol (v:v) based on the method of Folch, Lees and Sloane Stanley [2]. Thin layer chromatography, using 20×20 cm^2^ plates with a 60 Ǻ silica gel layer (Whatman International LTD, Maidstone, England), with a mobile phase of 60:40:2 heptane:diethyl ether: acetic acid (v:v:v) [3] was used to isolate phospholipids. Bands were visualized under UV light with 2,7-dichlorofluorescein (Sigma-Aldrich, Oakville, ON), and identified by comparison to a reference standard. Lipids were extracted off the silica using 2:1 chloroform:methanol with 22:3n-3 as an internal standard, and transesterified by 14% BF_3_ in methanol at 95 °C for 1 h to produce fatty acid methyl esters [4], which were separated by fast-gas chromatography [5], [6]. Peaks were identified by comparison of the retention time to a reference mixture of fatty acids (GLC-569, Nu-Chek Prep Inc). Fatty acids were quantitated by comparison to the internal standard and expressed as µg fatty acid/100 mg adipose tissue.

### 2.3 Statistical analysis

The data are expressed as mean±SD. Statistically significant differences between two groups were assessed by Student׳s *t*-test. Significance is inferred at *p*<0.05.

## Figures and Tables

**Table 1:** Adipose phospholipid fatty acyl composition from ad libitum fed versus fasted mice.

**Adipose phospholipid fatty acids (µg fatty acid/100 mg tissue)**		
**Name**	**Ad libitum**	**Fasted**
C 10:0	0.01±0.01	0.01±0.01
C 12:0	0.01±0.01	0.04±0.03
C 14:0	0.25±0.02	0.33±0.19
C 16:0	4.03±1.13	8.20±1.83⁎
C 18:0	6.54±2.22	11.51±4.24
C 20:0	0.38±0.08	0.59±0.40
C 22:0	0.17±0.10	0.16±0.07
C 24:0	0.13±0.07	0.16±0.06
**SFAs**	11.45±3.57	21.02±6.61⁎
C 12:1	0.01±0.01	0.04±0.04
C 14:1	0.00±0.01	0.02±0.03
C 16:1	0.14±0.06	0.21±0.10
C 18:1n-7	0.17±0.06	0.46±0.21⁎
C 18:1n-9	1.46±0.51	3.21±1.42
C 20:1n-9	0.10±0.03	0.18±0.16
C 22:1n-9	0.31±0.10	0.39±0.25
C 24:1n-9	0.08±0.05	0.20±0.03
**MUFAs**	2.26±0.70	4.69±1.75
C 18:2n-6	1.06±0.31	2.76±1.19⁎
C 18:3n-6	0.03±0.01	0.02±0.02
C 20:2n-6	0.04±0.04	0.13±0.08
C 20:3n-6	0.09±0.04	0.23±0.10⁎
C 20:4n-6	0.70±0.35	2.97±1.44⁎
C 22:2n-6	0.12±0.07	0.17±0.16
C 22:4n-6	0.09±0.04	0.23±0.17
C 22:5n-6	0.04±0.01	0.04±0.01
**N-6**	2.17±0.70	6.55±2.62⁎
C 18:3n-3	0.08±0.05	0.16±0.14
C 20:3n-3	0.07±0.01	0.12±0.14
C 20:5n-3	0.06±0.01	0.11±0.02⁎
C 22:5n-3	0.08±0.05	0.11±0.01
C 22:6n-3	0.39±0.10	0.73±0.09⁎
**N-3**	0.68±0.12	1.22±0.37⁎
**N-6/N-3**	3.22±1.01	5.92±3.16
**Total**	16.57±4.43	33.48±7.40⁎

*N*=3–4 mice per group, data are mean±SD. SFAs: total saturated fatty acids; MUFAs: total monounsaturated fatty acids; N-6: total n-6 polyunsaturated fatty acids; N-3: total n-3 polyunsaturated fatty acids.

⁎ *p*<0.05.

**Table 2:** Relative percent of fatty acyl species in adipose tissue phospholipids from ad libitum fed versus fasted mice.

**Adipose phospholipid fatty acids (% total fatty acids)**		
**Name**	**Ad libitum**	**Fasted**
C 10:0	0.07±0.05	0.02±0.03
C 12:0	0.08±0.05	0.11±0.07
C 14:0	1.25±0.26	0.91±0.34
C 16:0	22.85±1.55	23.38±0.15
C 18:0	37.08±4.74	32.27±5.49
C 20:0	2.23±0.23	1.60±0.78
C 22:0	1.13±0.60	0.46±0.17
C 24:0	0.64±0.31	0.46±0.13
**SFAs**	65.06±6.50	59.21±6.40
C 12:1	0.06±0.06	0.09±0.08
C 14:1	0.02±0.04	0.05±0.07
C 16:1	0.75±0.17	0.57±0.18
C 18:1n-7	0.92±0.32	1.40±0.73
C 18:1n-9	9.26±2.97	8.95±2.26
C 20:1n-9	0.52±0.15	0.50±0.37
C 22:1n-9	1.81±0.49	1.05±0.46
C 24:1n-9	0.63±0.51	0.59±0.21
**MUFAs**	13.97±3.50	13.18±2.43
C 18:2n-6	5.76±1.11	8.23±3.76
C 18:3n-6	0.13±0.09	0.07±0.06
C 20:2n-6	0.24±0.17	0.39±0.26
C 20:3n-6	0.48±0.34	0.70±0.37
C 20:4n-6	3.53±1.14	8.94±4.59⁎
C 22:2n-6	0.65±0.46	0.44±0.35
C 22:4n-6	0.53±0.33	0.73±0.69
C 22:5n-6	0.24±0.01	0.11±0.03⁎
**N-6**	11.57±2.80	19.61±8.76
C 18:3n-3	0.65±0.41	0.41±0.29
C 20:3n-3	0.45±0.15	0.30±0.30
C 20:5n-3	0.32±0.13	0.33±0.12
C 22:5n-3	0.47±0.26	0.32±0.04
C 22:6n-3	2.37±1.00	2.11±0.23
**N-3**	4.26±1.32	3.47±0.47
**N-6/N-3**	2.91±1.11	5.92±3.16

*N*=3–4 mice per group, data are mean±SD. SFAs: total saturated fatty acids; MUFAs: total monounsaturated fatty acids; N-6: total n-6 polyunsaturated fatty acids; N-3: total n-3 polyunsaturated fatty acids

⁎ *p*<0.05.

## References

[1] Marks K.A., Marvyn P.M., Henao J.J., Bradley R.M., Stark K.D., Duncan R.E. (2015). Fasting enriches liver triacylglycerol with n-3 polyunsaturated fatty acids: implications for understanding the adipose-liver axis in serum docosahexaenoic acid regulation. Genes Nutr..

[2] Folch J., Lees M., Sloane Stanley G.H.S. (1957). A simple method for the isolation and purification of total lipides from animal tissues. J. Biol. Chem..

[3] Christie W.W. (1989). Gas Chromatography and Lipids.

[4] Morrison W.R., Smith L.M. (1964). Preparation of fatty acid methyl esters and dimethylacetals from lipids with boron fluoride-methanol. J. Lipid Res..

[5] Stark K.D., Salem N. (2005). Fast gas chromatography for the identification of fatty acid methyl esters from mammalian samples. Lipid Technol..

[6] Metherel A.H., Aristizabal Henao J.J., Stark K.D. (2013). EPA and DHA levels in whole blood decrease more rapidly when stored at -20 degrees C as compared with room temperature, 4 and −75 degrees C. Lipids.

